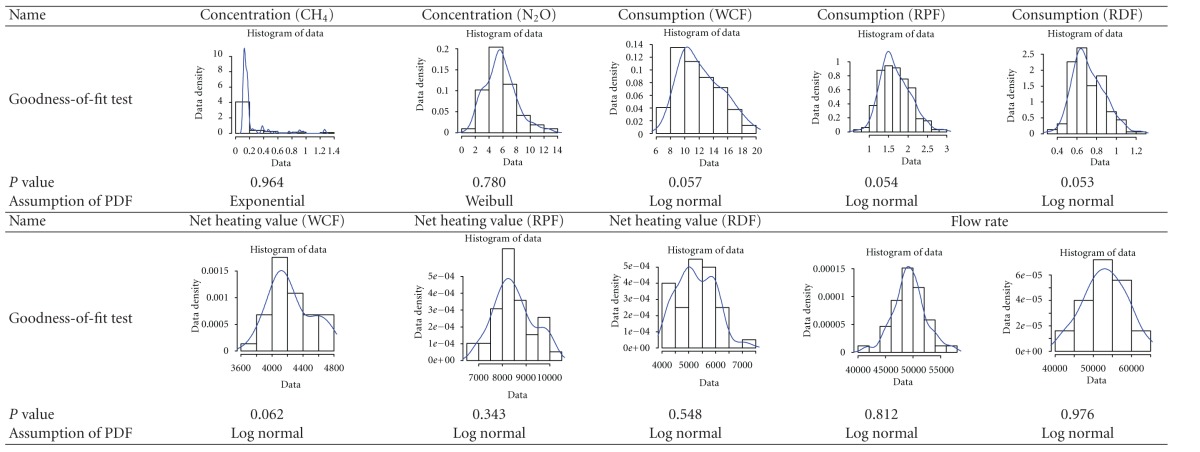# Development of Methane and Nitrous Oxide Emission Factors for the Biomass Fired Circulating Fluidized Bed Combustion Power Plant

**DOI:** 10.1100/2012/989242

**Published:** 2012-12-25

**Authors:** Chang-Sang Cho, Jae-Hwan Sa, Ki-Kyo Lim, Tae-Mi Youk, Seung-Jin Kim, Seul-Ki Lee, Eui-Chan Jeon

**Affiliations:** ^1^Cooperate Course for Climate Change, Sejong University, Seoul, Republic of Korea; ^2^Department of Environment and Energy, Sejong University, Seoul 143-747, Republic of Korea; ^3^Department of Statistics, Korea University, Seoul, Republic of Korea

## Abstract

This study makes use of this distinction to analyze the exhaust gas concentration and fuel of the circulating fluidized bed (CFB) boiler that mainly uses wood biomass, and to develop the emission factors of Methane (CH_4_), Nitrous oxide (N_2_O). The fuels used as energy sources in the subject working sites are Wood Chip Fuel (WCF), RDF and Refused Plastic Fuel (RPF) of which heating values are 11.9 TJ/Gg, 17.1 TJ/Gg, and 31.2 TJ/Gg, respectively. The average concentrations of CH_4_ and N_2_O were measured to be 2.78 ppm and 7.68 ppm, respectively. The analyzed values and data collected from the field survey were used to calculate the emission factor of CH_4_ and N_2_O exhausted from the CFB boiler. As a result, the emission factors of CH_4_ and N_2_O are 1.4 kg/TJ (0.9–1.9 kg/TJ) and 4.0 kg/TJ (2.9–5.3 kg/TJ) within a 95% confidence interval. Biomass combined with the combustion technology for the CFB boiler proved to be more effective in reducing the N_2_O emission, compared to the emission factor of the CFB boiler using fossil fuel.

## 1. Introduction

The greenhouse gas emissions causing global warming have been consistently rising since the Industrial Revolution, increasing up to 70% between 1970 and 2004 worldwide. If this trend continues, the average temperature of the Earth by the end of the 21st century is expected to rise by up to 6.4°C [1], compared to present averages. To resolve this problem, each country in the world is establishing and implementing national plans to lower greenhouse gas emission. As a part of the efforts, the global community ratified the Kyoto Protocol at the Conference of the Parties, held in Kyoto in 1997. The protocol suggested 5.2% of reduction in greenhouse gas emission between 2008 and 2012, the first targeting period, compared to that of 1990 [2]. Korea also announced 30% reduction target compared to Business As Usual (BAU) by 2020, to reduce greenhouse gas emission. This target is quite a forward-looking goal for a non-Annex country of the Kyoto Protocol that is not required to lower emission [3].

To effectively achieve the reduction goal, the most essential aim is the figuring out of the national emission of greenhouse gas and developing emission inventory based on rational data. As for non-CO_2_ emission, the global warming potential (GWP) of methane (CH_4_) and nitrous oxide (N_2_O) is 21 and 310 times that of carbon dioxide (CO_2_), respectively, which should not be overlooked in developing the inventory. The emission characteristics of the non-CO_2_ greenhouse gas fluctuate according to several factors such as type of emission source, type and property of fuel, form of boiler, type of prevention facility, and load factor [4, 5].

Therefore, Intergovernmental Panel on Climate Change (IPCC) recommends the application of country-specific or technology-specific emission factors instead of the Tier 1 default emission factor, when each country calculates its own greenhouse gas emission [6]. However, most R&D on emission factors of energy sector both domestic and international was carried out for CO_2_ rather than non-CO_2_ [7, 8].

This study develops the emission factor of non-CO_2_ gas released from combined heat and power plants that use biomass. Using biomass as an energy source does not influence the increase of CO_2_ in the air because the CO_2_ absorbed in the process of photosynthesis goes back to the air when the biomass is used [9]. The biomass substituting fossil fuel has the effect of lowering the greenhouse gas emission by the increase in the emission that would have generated due to the combustion of fossil fuel [10]. However, the emission of non-CO_2_ such as CH_4_ and N_2_O is not excluded in the report on the total greenhouse gas emission. In addition, a preliminary research revealed that the N_2_O emission of the fluidized bed boiler is quite high when compared to that of the coal-fired boiler [11]. Therefore, developing non-CO_2_ emission factor of biomass-using circulating fluidized bed (CFB) power plants, which is appropriate for the domestic circumstances would ensure very important basic data for the estimation and forecast of greenhouse gas emission and emission reduction plans.

## 2. Designation of Subject Facilities

Fluidized bed combustion technology is one of the most simple and environmentally friendly ways to combust solid fuel such as coal. The technology enables the temperature maintenance of the combustion furnace at 900°C, prohibiting the creation of nitrogen oxide and directly desulfurizing inside the furnace with lime stone. The technology also enables mixed combustion of various solid fuels such as biomass and wood chip while combusting coal [12–15]. There have been consistent efforts to ensure that the technology enables fuel diversification through mixed combustion of multicarbon coal and mixed combustion of coal and other fossil fuels, and the combustion which is economically feasible and environmentally friendly.

This research selected Plant B that uses biomass as a main fuel instead of fossil fuel. The working site of Plant B operates a 50 MW CFB power plant. Wood chip fuel (WCF) accounts for about 70% of the total fuel. Furthermore, 20% of the fuel is refused plastic fuel (RPF) which is made by processing waste synthetic resins such as vinyl into pellet type. 10% of the fuel is refused derived fuel (RDF) which is made by grinding, compressing, and molding daily wastes, creating pellet type fuel. The working site is using 22,677 ton/month of alternative fuel which is the largest compared to those of other working sites using alternative fuel except waste tires. Detailed information of using alternative fuels is shown in Table 1. 

## 3. Research Method

### 3.1. Analysis Method of Energy Source

We collected about 1 kg of fuel sample when the fuels were put into the facility. The collected sample was ground with a rotating grinder. First, the sample was ground to pass a screen with 4.75 mm of gradation, which is coarse grinding. Second, the sample was ground to pass a screen with 1 mm of gradation, which is fine grinding. Then, the ground sample was treated with the increment reduction method. The prepared sample was used to analyze its heating value. The heating value analyzing equipment used in this study was an automatic calorimeter (IKA-C2000, Germany). The equipment measures the temperature increase during the combustion of the sample, calculating the heating value per 1 g of sample. The heating values of energy sources are divided into gross calorific value (GCV) and net calorific value (NCV). Gross caloric value is the heating value generated during the combustion of the sample which includes the vapor and the latent heat in the combustion gas. Meanwhile, NCV excludes the vaporization heat of moisture generated during the combustion of moisture or hydrogen in the fuel. This research converted the dry-basis GCV into an NCV which is suggested by IPCC 2006 G/L, by applying the content of hydrogen and moisture.

### 3.2. Method of Collecting Exhaust Gas Sample

In order to confirm the emission characteristics of CH_4_ and N_2_O that are released from the emission source, field surveys such as the analysis of exhaust gas flow, moisture amount, and gas sample were performed. The field survey company measured the temperature of the exhaust gas, moisture amount, air temperature, flow velocity, and pressure when it collected samples of greenhouse gas [16, 17].

The temperature of the exhaust gas of the greenhouse gas emission facilities is above 100°C in general. Therefore, the sampling tubes and sample collecting pipes should be made of materials that can stand high temperatures and flow velocities. So, we manufactured sampling tubes made of stainless steel that can withstand high temperature and flow velocity, satisfying the Koran Standard Method (7 mm of inner diameter). The length of the tube is 1.5 m. Two tubes are linked when the inner wall or inner diameter of the stack is big. To remove the moisture included in the exhaust gas, we installed a moisture absorption bottle with silica gel in front of the equipment collecting exhaust gas so that the greenhouse gas sample did not include moisture. The moisture collecting equipment was also made of the same material as that of the sampling tube. To prevent the clogging of the filter medium caused by corrosion of sampling tube or pipe or moisture condensation, we heated the whole sampling tube with the electric heating method up to 120°C, collecting moisture included in the exhaust gas with anhydride calcium hydride.

**Figure 1:**
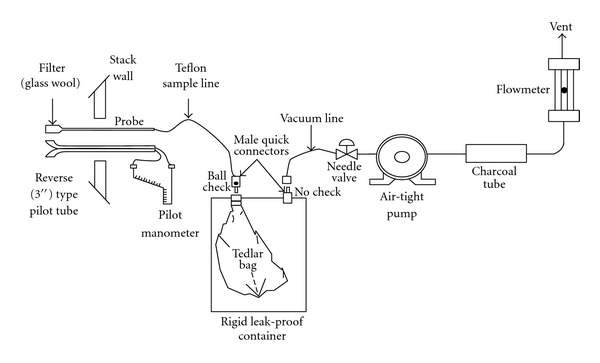
Greenhouse gas sampling using lung sampler.

This research used 10 L of Tedlar bag when collecting exhaust gas samples, applying EPA method 18 [18]. The diagram is provided in Figure 1. To collect representative samples, we collected samples with 20 to 30 minutes of interval at the emission source (stack) from 10 AM to 6 PM for a period of five days.

### 3.3. Method of Analyzing the Concentration of Non-CO_2_ Greenhouse Gas

We covered the light over collected samples and analyzed at a laboratory on the day of collecting. CH_4_ in the exhaust gas was analyzed with Gas Chromatography with Flame Ionization Detector (GC-FID). CH_4_ is known as a representative compound of hydrocarbon that is analyzed with GC-FID [19]. N_2_O was analyzed with gas chromatography and its concentration was analyzed with the electron capture detector (ECD). GC-ECD is a widely used detector for N_2_O and chlorofluorocarbons (CFCs).

We used 1 m and 3 m of Porapak Q 80/100 mesh packed column (Stainless steel, 3.175 mm external diameter, Restek) and ultrapure nitrogen as carrier gas. The flux of the carrier gas was set at 20 mL/min and the temperatures of the injection port and oven were set at 120°C and 70°C, respectively. To remove oxygen and moisture in the gas sample, we used 10, 6, and 4 ports of switching valve. The detector was set at 320°C. Detailed condition of the analysis is as shown in Table 2.

**Figure 2:**
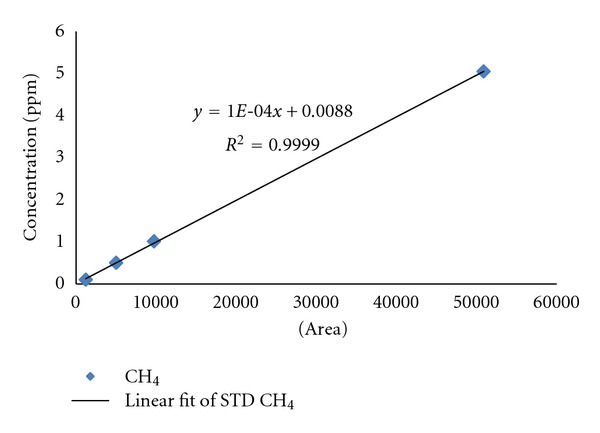
Calibration curve by CH_4_ standard.

To conduct a quantitative analysis, we used 0.1, 0.5, 1.0 and 5.0 ppm of standard gas, drawing a calibration curve. The calibration curve showed a sound linearity which is *R* ^2^ = 0.9999.  Figure 2 was used in analyzing the concentration of the collected sample.

**Figure 3:**
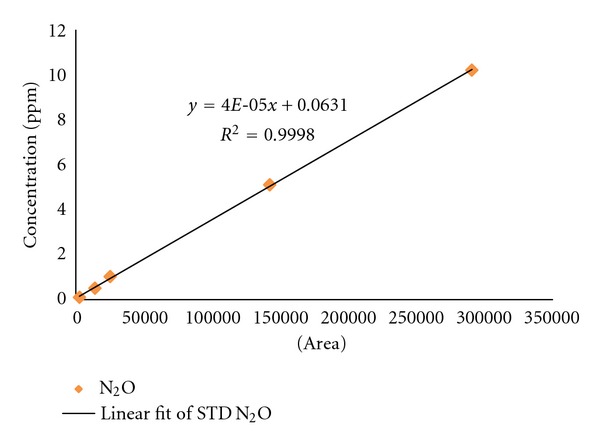
Calibration curve by N_2_O standard.

To conduct a quantitative analysis, we used 0.1, 0.5, 1.0, 5.0, and 10.2 ppm of standard gas, drawing a calibration curve. The calibration curve showed a sound linearity which is *R* ^2^ = 0.9998.  Figure 3 was used in analyzing the concentration of the collected sample.

### 3.4. Method of Calculating the Emission Factor of Non-CO_2_

As for CO_2_, highly reliable emission factors can be calculated with the element analysis of the fuel. However, the emission factor of non-CO_2_ is affected by combustion condition such as combustion technology. Therefore, the emission factor calculated based on the fuel analysis may not be used as a representative value. So, this study measured the CH_4_ and N_2_O in the exhaust gas of the CFB power plant that uses biomass as a main fuel. The worksheet of the emission factor is comprised of 5 steps, which are shown in Table 3.

First, input the contents of carbon and hydrogen that were calculated in analysis of fuel. Second, measure a GCV, and to convert GCV into NCV, applying the result of the element analysis conducted in step 1. Moreover, input the data for consumption of fuel in the field. Third, input measured concentrations of CH_4_ and N_2_O in the exhaust gas. Fourth, calculate the amount of CH_4_ and N_2_O. Fifth, calculate emission factors of CH_4_ and N_2_O.

## 4. Result and Consideration

### 4.1. Accuracy Management of the Analyzing Equipment

Table 4 shows the result of the reproducibility and the method detection limit (MDL) which is needed for the analysis accuracy management of CH_4_ and N_2_O, the study subject of this paper. To evaluate the reproducibility, we analyzed the standard gases which are 1.01 umol/mol of CH_4_ and 1.02 umol/mol of N_2_O, three times repetitively. As for the reproducibility, the standard error (SE) and the relative standard error (RSE) of CH_4_ and N_2_O were 18.15 and 0.18%, and 125.01 and 0.291%, respectively. We also measured the detection limit of at least seven samples of which concentration of the measuring item is high enough to be detected. The detection limit was calculated by multiplying the standard deviation of repetitive measurements and the distribution value *t* of the freedom degree. Distribution value 3.143, which is for the degree of freedom 6, was applied within 98% of reliability in the repetitive measuring of seven samples [20]. As a result, the detection limits of CH_4_ and N_2_O were 0.013 ng and 0.074 ng, respectively.

### 4.2. Fuel Characteristics of Subject Power Plant

#### 4.2.1. Analysis Result of Heating Value

The production and consumption of solid, liquid, and gas fuel is expressed with a physical unit such as tons in the statistics or data of energy. The heating value is needed to convert the unit into another energy unit a Joule. This study calculated each energy source's as received basis (ABS) net heating value that is used in the 2006 IPCC Guideline.

The subject working sites use wood chips made of waste wood, RDF that are made of daily wastes through grinding, compression, and molding, and RPF which is a pellet-type fuel processed from waste synthetic resins such as vinyl and plastic, as energy sources. This study collected the samples on separate dates to increase the reliability. We analyzed eight samples of each energy source. The results are shown in Table 5.

The ARB NCV of RDF, RPF, and WCF are 11.9 TJ/Gg (RSD 10.1%), 17.1 TJ/Gg (RSD 28.9%), and 31.2 TJ/Gg (RSD 17.9%), respectively. The RPF of vinyl or plastic with high heating value was the highest. The RSD of the heating value of RDF-5 or RPF was more than 20% higher than others. This is because the composition rarely remained steady when the fuel was made of wastes.

#### 4.2.2. The Result of Element Analysis

The combustion characteristics are greatly affected by the main elements of the fuel such as carbon (C), hydrogen (H), nitrogen (N), sulfur (S), and moisture (including adherent moisture and inherent moisture), ash, volatile component, and adherent carbon. In particular, C is involved in the generation of CO_2_, one of greenhouse gases, and used in calculating the heating value of the vapor created due to hydrogen. Therefore, this study analyzed the content of carbon and hydrogen in the fuel, by analyzing the elements of the energy sources used in the subject working sites. The results are shown in Table 6. Carbon and hydrogen account for 44% (RSD 4.4%) and 5%, 46% (RSD 13.9%) and 6%, and 67% (RSD 16.7%) and 8% of the WCF, RDR-5, and RPF, respectively. The results show that there is big gap between RDF and RPF, compared to WCF.

### 4.3. Emission Characteristics of Non-CO_2_

Table 7 shows the analysis results of emission concentration of CH_4_ and N_2_O that are measured at CFB power plants using biomass and alternative fuel. The average concentration of CH_4_ of the exhaust gas is 2.78 ppm, with the minimum and maximum concentration ranges of 0.1 ppm and 3.27 ppm, respectively. The analysis of 104 samples showed that the SE of CH_4_ is 0.02. The average concentration of CH_4_ is 7.68 ppm, with the minimum and maximum ranges of 1.15 ppm and 12.75 ppm, respectively. The average error is 0.22 in the analysis of 108 samples. As for the emission characteristics of the CH_4_ and N_2_O, those of CH_4_ rarely changed at low concentration. 

The emission concentration is lower than that of the power plants using fossil fuel. This is because the fuel is mixed well with the help of CFB, unlike the general combustion method. The emission concentration of N_2_O is rather higher than that of power plants using fossil fuel. The combustion of CFB power plants is conducted at the relatively low temperature of 900°C, unlike that of the fire-powered method. Previous studies indicated that NOx reduces at this temperature, while the emission of N_2_O rises (e.g., [2]).

### 4.4. Calculation of Emission Factor of Non-CO_2_

Table 8 showed that the analysis result of exhaust gas of subject facilities, energy sources, and the emission factor of CH_4_ and N_2_O that are calculated with the process data. The emission factor of CH_4_ of CFB power plant using biomass as a main energy source was calculated to be 1.2 kg/TJ which is within the same range of emission factor suggested by 2006 IPCC Guideline. The emission factor of N_2_O is 3.3 kg/TJ which is lower than that suggested by IPCC. Like other studies, this research also showed that the emission concentration of N_2_O could be reduced more when the CFB power plant uses biomass or other fuel mixed with biomass than when it uses 100% fossil fuel [21, 22]. The IPCC suggested 61 kg/TJ and 7 kg/TJ as the basic emission factor of N_2_O released from the CFB boilers using just bituminous coal and peat, respectively. There is gap between the actual emission factor and the basic emission factor. Unlike the emission factor of CO_2_ of adherent combustion, those of non-CO_2_ such as CH_4_ and N_2_O are greatly affected by type, size, and mixing rate of combustion and the combustion technology of boilers.

### 4.5. Uncertainty Assessments

To produce a more reliable average value in developing a final emission factor, we used a probability method of average forecasting that is suggested by 2006 IPCC G/L. This research used Monte Carlo simulation among other forecasting methods. The Monte Carlo analysis is useful when in the detailed sector assessment of uncertainty, the uncertainty is high, distribution is irregular, algorithm is complex functions, and there is a correlation between some activities group and emission factors.

In the Monte Carlo simulation, probability density function (PDF) should be designated to rationally represent each model of uncertainty. To designate it, we carried out the goodness-of-fit test of the probability density function.

The goodness-of-fit test is the statistical inference to find out whether the sample distribution has the theoretical distribution. The null hypothesis is that the observed data comes from a specific distribution. Rejecting the null hypothesis means that the data distribution does not follow the theoretical distribution. The criterion of the rejection is determined by significant level *α* that is the maximum allowable limit of probability fallible in process of the hypothesis test. The null hypothesis is rejected when the significant probability (*P* value), which is calculated by a test statistic, is less than a significant level. The test statistics used in this study are Kolmogorov-Smirnov (K-S) and Anderson-Darling (A-D). The K-S test is based on the empirical distribution function (ECDF). *D* is calculated as follows:

(1) $$\begin{array}{c} D=\underset{1\leq i\leq N}{\max}\left(F\left(Y_{i}\right)-\frac{i-1}{N},\frac{i}{N}-F\left(Y_{i}\right)\right), \end{array}$$

where *Y*_1_, *Y*_2_,…, *Y*_*N*_ are *N*-ordered data points, and *F* is the theoretical cumulative distribution.

K-S test has an advantage in which *D* is not affected by a cumulative distribution function of the theoretical distribution and it is an exact test. Meanwhile, it tends to be more greatly affected by the center than the tail in the distribution. In addition, the parameters of the distribution should be fully specified.

A-D statistic could be used in several distributions where weak points of the K-S should be considered, and adds more weight on the tail than the K-S does. The test statistic, *A* ^2^, is as follows:

(2) $$\begin{array}{c} A^{2}=-N-S,\\ S=\sum_{i=1}^{N}\frac{\left(2i-1\right)}{N}\left[\ln F\left(Y_{i}\right)+\ln\left(1-F\left(Y_{N+1-i}\right)\right)\right], \end{array}$$

where *Y*_1_, *Y*_2_,…, *Y*_*N*_ are *N*-ordered data points, and *F* is the theoretical cumulative distribution.

**Figure 4:**
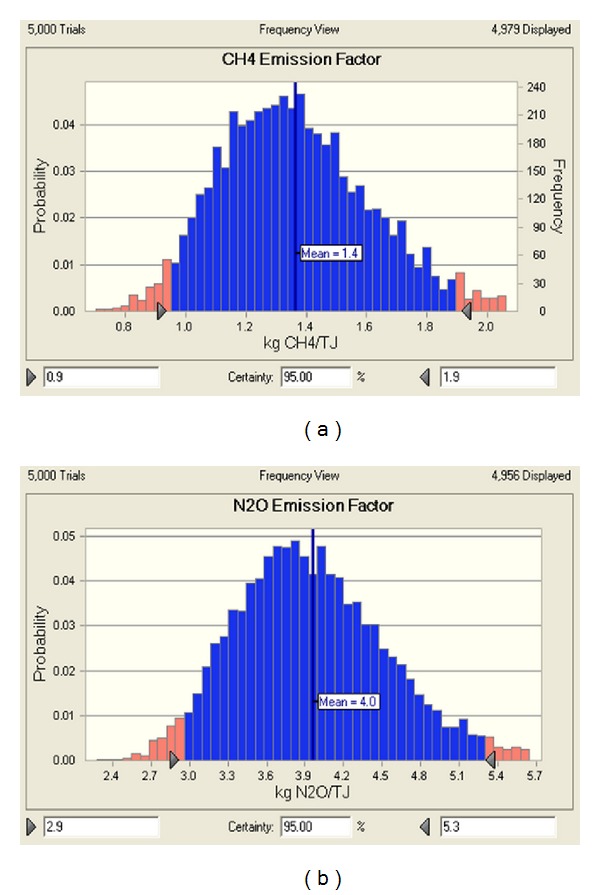
The result of Monte Carlo simulation on CH_4_ and N_2_O emission factors.

All tests were realized by applying statistical program “*R*” (ver. 2.12.0) and the significant level was 5%. We determined the probability density function by a goodness-of-fit test of emission concentration of CH_4_ and N_2_O, emission flux, fuel consumption, and low heating value of the fuel which are the data necessary to calculate the emission factors of CH_4_ and N_2_O. The result is shown in Table 9. The data set was randomly generated 5,000 times from the appropriate probability density function using the Cristal Ball software of Monte Carlo simulation. The result is shown in Figure 4. The emission factors of CH_4_ and N_2_O that are calculated in the Monte Carlo simulation are 1.4 kg/TJ (0.9–1.9 kg/TJ) and 4.0 kg/TJ (2.9–5.3 kg/TJ), respectively, within the reliability range of 95%.

## 5. Result

This study analyzed the fuel of CFB boiler using wood biomass as a main fuel with a percentage of 70%, measuring the concentration of CH_4_ and N_2_O in the exhaust gas to calculate their emission factors. The subject working sites use WCF, RPF, and RDF as fuels. The fuel analysis showed that the ARBs of WCF, RDF, and RPF are 11.9 TJ/Gg (RSD 10.1%), 17.1 TJ/Gg (RSD 28.9%), and 17.9 TJ/Gg (RSD 17.9%), respectively. The heating value of the RPF using vinyl and plastic of which heating value is high was the highest. The concentration analysis showed that the average concentration of CH_4_ is 2.78 ppm within the minimum and maximum ranges of 0.1 ppm and 3.27 ppm, respectively. The SE of the average concentration is 0.02, which is the result of analyzing 104 samples. In addition, the average concentration of N_2_O is 7.68 ppm within the minimum and maximum ranges of 1.15 ppm and 12.75 ppm, respectively. Its SE is 0.22, which is the result of analyzing 108 samples.

We used the analysis result and the data collected in the field survey to calculate the emission factor of CH_4_ and N_2_O of the CFB boiler using biomass. The calculated emission factor of CH_4_ was 1.4 kg/TJ (0.9–1.9 kg/TJ) within the reliability range of 95%, which is in the same range as the basic emission factor suggested by 2006 IPCC Guideline. The emission factor of N_2_O was 4.0 kg/TJ (2.9–5.3 kg/TJ) within the reliability range of 95%. The emission factor is lower than that suggested by IPCC. This is because unlike the emission factor of CO_2_ in the adherent combustion, those of non-CO_2_ such as CH_4_ and N_2_O are greatly affected by type, size, and mixture portion of fuel, and the combustion technology of the boiler.

This study compared the IPCC emission factor and the emission factor calculated in this research, which indicated that the combustion technology of CFB boiler using biomass could greatly reduce N_2_O emission, compared to that of fossil fuel.

## Figures and Tables

**Table 1:** Plant lists of using alternative fuels.

	Plant name	The amount of alternative fuels (ton/month)	Type of fuels
1	Plant A	27,097	TDF*
2	Plant B (study site)	22,677	WCF, RDF, and RPF
3	Plant C	10,722	RPF
4	Plant D	8,572	RPF
5	Plant E	5,996	RPF
6	Plant F	4,227	RPF

*TDF: tire derived fuel.

**Table 2:** Analysis condition of GC for N_2_O and CH_4_.

	GC/FID	GC/ECD
Column	Porapack Q 80/100	Parapack Q 80/100
Carrier gas	N_2_ (99.999%)	N_2_ (99.999%)
Flow	30 mL/min	20 mL/min
Temperature		
Oven	80°C	70°C
Injector	100°C	120°C
Detector	250°C	320°C
Detector range	0	0

**Table:** (a) Step 1 (fuel data)

Item	Carbon of fuel (as received basis)	Carbon of fuel (air-dried basis)	Carbon of fuel (dry basis)	Inherent moisture	Total moisture	Hydrogen
Subitem			*①*	*②*	*③*	*④*
Unit	(%)	(%)	(%)	(%)	(%)	(%)
Calculation	*①*× ((100 −*②* ) ÷ 100)	*①*× ((100 −*③* ) ÷ 100)				

**Table:** (b) Step 2 (raw data)

Item	Gross heating value	Net heating value	Fuel consumption rate	Heating output
Subitem	*A*	*B*	*C*	*D*
Unit	(kcal/kg)	TJ/ton	ton/h	TJ/h
Calculation		([*A* − {600 × (9 ×*③*+*④* )}] × 4.18) × 10^−6^		*B* × *C*

**Table:** (c) Step 3 (non-CO_2_ concentration)

Item	Volume concentration	Mass concentration	Flow rate
Subitem	*E*	*F*	*G*
Unit	ppm	mg/m³	m³/h
Calculation		*E* × (16 or 44 ÷ 22.4)	

**Table:** (d) Step 4 (non-CO_2_ emission) and Step 5 (non-CO_2_ emission factor)

Item	Non-CO_2_ emission	Non-CO_2_ emission factor
Subitem	*H*	*I*
Unit	g/h	kg/TJ
Calculation	*F* × *G* ÷ 10^³^	*H* ÷ *D*

**Table:** (a) Results of reproducibility

Substance	Result of reproducibility test (area)			Statistical data		
	1	2	3	Mean	SE^a^	RSE (%)
CH_4_	9,775	9,767	9,717	9,753	18.15	0.186
N_2_O	24,689	24,939	24,811	24,813	125.01	0.291

^a^Standard error (SE): standard deviation.

**Table:** (b) MDL

Classification		1	2	3	4	5	6	7	Mean	SD^b^	MDL ng
CH_4_	Peak area	724	690	764	642	655	705	679	694	41	0.013
N_2_O	Peak area	3,232	2,684	2,574	3,655	2,221	1,612	2,574	2,650	661	0.074

^b^SD: standard deviation.

**Table 5:** Caloric value of alternative fuels for combustion.

Sample name	Gross calorific value (TJ/Gg)	Net calorific value (TJ/Gg)	RSD (%)	Moisture (%)	Number of sample (EA)
WCF	13.7	11.9	10.1	25.1	8
RDF	19.0	17.1	28.9	24.2	8
RPF	33.5	31.2	17.9	10.0	8

**Table 6:** Elementary analysis of various fuels.

Sample name	C (%)	H (%)	Number of sample (EA)
WCF	44.0	5.6	8
RDF	46.5	6.0	8
RPF	66.8	8.5	8

**Table 7:** Average value of CH_4 _and N_2_O concentration in the biomass fired circulating fluidized bed power plants.

Sample	Mean	SD	Min.	Max.	SE^a^	*N*
Methane	2.78	0.22	0.10	3.27	0.02	104
Nitrous oxide	7.68	2.25	1.15	12.75	0.22	108

^a^Standard error (SE): standard deviation/$\sqrt{N}$.

**Table 8:** Compare with emission factors of circulating fluidized bed boiler.

	Type of fuels	Emission factors (kg/TJ energy input)	
		CH_4_	N_2_O
This study	WCF, RDF, and RPF	1.2	3.3
2006 IPCC	Bituminous	1	61
	Peat	3	7

**Table 9:** Assumption of probability distribution.